# The implementation of “integration of sports and medicine” in China: Its limitation and recommendations for model improvement

**DOI:** 10.3389/fpubh.2022.1062972

**Published:** 2022-12-22

**Authors:** Ting Zhang, Zhihao Ning, Li Dong, Song Gao

**Affiliations:** ^1^Department of Sports and Human Science, College of Physical Education and Health Sciences, Zhejiang Normal University, Jinhua, China; ^2^Department of Traditional Chinese Medicine, University Hospital, Zhejiang Normal University, Jinhua, China; ^3^Department of Chinese Medicine, Naval Special Medical Center, Naval Medical University, Shanghai, China

**Keywords:** integration of sports and medicine, Healthy China 2030, policy recommendations, Suzhou Model, Shanghai Model, limitations

## Abstract

The “integration of sports and medicine” (ISM) under the “Healthy China 2030” strategy can alleviate the contradiction between residents' health needs and medical supply. Under the “Healthy China 2030” strategy, the government-initiated calls and measures for the integration of sports and medical institutions based on the actual region conditions. This article outlines the “Sports-Medical Integration” program implemented in the coastal cities of China's southeast, Suzhou and Shanghai. We described the specific implementation modes and related deficiencies of the ISM, taking Suzhou and Shanghai as examples. Through three policy recommendations, we put forward the idea of promoting the development of a new model of ISM in China.

## Introduction

The comprehensive model of sports combined with medical services plays an active role in chronic disease prevention, rehabilitation, and health promotion (1). As early as the 19th century, American health professionals tried to combine sports and medicine, and explored its feasible implementation mode (2). Both sports and medicine serve public health. Sports improve physical function and prevent diseases through exercise; medical services focus on prevention, treatment, and rehabilitation. The proper combination of the two is an effective way to improve public health and promote the recovery of chronic diseases (3). Physical fitness assessment and the health status of medical examinations are cross-referenced to formulate scientific and personalized exercise prescriptions to solve health problems.

At present, China's population is aging, and the incidence of chronic diseases is increasing year by year. Society and the government are facing major public health challenges (4, 5). The government issued policies aimed at improving citizens' health and effectively controlling the development of chronic diseases. The outline of the “Healthy China 2030” plan and China's Medium- and Long-term Plan for Prevention and Treatment of Chronic Diseases (2017–2025) mentioned that the ISM will be the inevitable trend of serving public health and preventing chronic diseases in the future (6, 7). After the Beijing Olympics, the country's sports industry development goal has gradually shifted from “Olympic medal fever” to promoting public health. The promulgation of policies such as the 13th Five-Year Plan for sports development, the National Human Rights Action Plan of China (2016–2020), and other policies have made it clear that the sports industry has begun to tilt toward national health (6, 8, 9). This has promoted the formation of disease management and health service model combining sports and medicine. The ISM has been elevated to an important way to create a healthy lifestyle for citizens.

The Several Opinions of the State Council on Accelerating the Development of the Sports Industry and Promoting Sports Consumption put forward the task requirements for promoting integrated development such as “promoting the integration of the sports industry and other industries,” and “actively expanding business formats, promoting the integration of sports and medicine, and encouraging mutual accommodation” (10). In the outline of the “Healthy China 2030” Plan, “sharing and co-construction” is the strategic theme of building a healthy China (6). The “Healthy China 2030” made a strategic plan for the integration of the sports industry into the health industry, and specifically discussed the themes of developing new forms of health services, cultivating the sports medical rehabilitation industry and actively developing the fitness and leisure sports industry (6). At present, the ISM is facing unprecedented opportunities: a series of policies are issued to point out the direction for the ISM; industrial integration opens a new path for the ISM; people's multi-level and diversified health needs provide an impetus for the ISM. The sports industry is an important part of the health industry, and it is also an inevitable trend to integrate and develop with related industries and to innovate in business formats. The policies and documents related to the ISM are shown in Table 1.

**Table 1:** Policies or documents related to the ISM.

**File name**	**Main contents**
Several Opinions of the State Council on Promoting the Development of the Health Service Industry. Beijing: General Office of the State Council (2013) (24)	“Guide relevant universities to reasonably determine the scale of training relevant professional talents… standardize and accelerate the training… rehabilitation therapists, health managers, fitness coaches, social sports instructors and other practitioners”
Several Opinions of the State Council on Accelerating the Development of the Sports Industry and Promoting Sports Consumption. Beijing: General Office of the State Council (2014) (10)	“Promoting the integration of the sports industry and other industries,” and “actively expanding business formats, promoting the integration of sports and medicine, and encouraging mutual accommodation”
Outline of the “Healthy China 2030” Plan. Beijing: General Office of the State Council (2016) (6)	“Grasp the law of health development, adhere to prevention first”
The 13th Five-Year Plan for sports development. Beijing: General Administration of Sport (2016) (8)	“The sports industry inclines to the health of the whole people”
National Human Rights Action Plan of China (2016-2020). Beijing: General Office of the State Council (2016) (9)	“National fitness is an important way to achieve national health and a basic guarantee for all people to build up their health and lead a happy life”
China's Medium- and Long-term Plan for Prevention and Treatment of Chronic Diseases (2017-2025). Beijing: General Office of the State Council (2017) (7)	“The ISM will be the inevitable trend of serving public health and preventing chronic diseases in the future”
Opinions of the State Council on implementing the Healthy China Action Plan. Beijing: General Office of the State Council (2019) (15)	“People's health is an important symbol of the prosperity of a nation, and prevention is the most economical and effective health strategy”
Opinions of the State Council on Promoting the inheritance and innovation of traditional Chinese medicine. Beijing: General Office of the State Council (2019) (12)	“Promote the integration of traditional Chinese medicine, traditional Chinese sports and modern rehabilitation technology, and develop rehabilitation medicine with Chinese characteristics”
Outline of the “Healthy China 2030” Plan. Beijing: General Office of the State Council (2021) (16)	By 2025, the national fitness public service system will be improved, and people will enjoy more convenient sports and fitness, with 38.5 percent of people regularly taking part in physical exercise

Since the government issued the “Healthy China 2030” policy in 2016, the awareness of exercise to promote health has gradually spread among the public. However, there is no unified application mode of physical medicine in different provinces and cities. Many intervention studies focused on exercise improving disease symptoms, such as aerobic exercise improving blood sugar and insulin resistance in type 2 diabetes (11). The benefits of traditional Chinese sports on diseases are gradually being discovered (12). Under the background of the ISM, universities, especially those with sports related majors, focus on combining theory with practice to cultivate social sports instructors with comprehensive skills (13, 14). However, sports instructors lack professional certification, which is detrimental to their career development. Very few hospitals offer “exercise prescription” services because doctors are not experts in exercise. Doctors need the sports instructors' assistance, who are unable to work in hospitals. This is the key to the ISM is difficult to achieve. The ISM services led by local governments is mainly aimed at raising residents' health awareness and encouraging them to participate in fitness activities (15, 16). At present, the development mode of the ISM in China is shown in Table 2.

**Table 2:** The development mode of the ISM in China.

**Model**	**Illustration**	**Details**
Community mode	Culture and Sports Service Center, Malu Town, Jiading District, Shanghai; National Physique Monitoring Station, Lucheng District, Wenzhou City, Zhejiang Province	Guided by the government and based on the community, it conducts residents' physical fitness monitoring, sports risk prevention, fitness guidance and health management.
Fitness club mode	Suzhou Sunshine Fitness Card program; UT Sports Clinic in Hefei	Medical rehabilitation institutions shall be established in sports venues. Make full use of the knowledge of medicine, sports and rehabilitation to evaluate the physical fitness of all members and put forward the corresponding exercise prescription.
Hospital mode	Beijing Beitaipingzhuang Hospital; Beijing Medical Examination Center	Special institutions have been set up in hospitals to carry out physical monitoring, disease prevention, and other work for chronic patients.
The combination of industry, university and research	Shenzhen Taishan Sports Technology Co., LTD.; Institute of Population, Peking University	Scientific research institutions cooperate with hospitals to carry out experimental attempts of physical and medical integration.
Multi-agent co-construction mode	Qingdao Institute of Integrative Medicine (ISM)	With first-class hospitals, research institutes and associations as multiple subjects and market-oriented, the construction of an innovation system will be promoted for the big health industry that integrates physical and medical services.

The ISM is an important means to alleviate the contradiction between national health demand and medical supply. According to 2013 survey data: “Compared with 2008, the prevalence of chronic diseases in China has increased by 9%,” which is equivalent to an increase of 120 million patients (17, 18). The rapid increase in the number of patients with chronic diseases has taken up a lot of medical and health resources. Medical and health institutions cannot effectively divert people's growing medical needs, which deepens the contradiction between supply and demand. This proactive, low-cost, and long-benefit “sports + medical” model promotes health to most people, thereby reducing the medical supply of patients and doctors. The ISM can divert some of the treatment places of chronic patients from hospitals to fitness places, to achieve the goal of optimizing medical resources, alleviating conflicts between doctors and patients, and reducing financial burdens.

## Policy options and implications

### Suzhou Model: A new attempt of the “Sunshine Fitness Card” policy

In 2005, the Suzhou Municipal Government formulated the “Sunshine Fitness Card” policy and issued the Notice on Doing a Good Job in Applying for the “Sunshine Fitness Card” for Medical Insurers. This is the first nationwide use of a combination of sports and medical care. Applicants can transfer the balance of their medical insurance account to the “Sunshine Fitness Card” as required for sports consumption in designated sports and fitness centers. In terms of transfer standards, the Suzhou Social Security Office has issued several standards for the transfer of amounts for applicants to choose from. According to the balance of the medical insurance account and combined with their exercise habits, the claimant can choose four types of personal account funds of 500 RMB, 1,000 RMB, 1,500 RMB, and 2,000 RMB to transfer to the special account of the “Sunshine Fitness Card” at one time.

So far, Suzhou has introduced the “Sunshine Fitness Card,” and the number of people applying for the card and the transfer amount has been increasing year by year. In 2006, the number of people who applied for the “Sunshine Fitness Card” and the transfer amount were 1,116 and 67,500 RMB respectively; In September 2013, the cumulative number of people applying for a card in the urban area was 35,372, and the amount of 38,434,200 RMB in the medical insurance account was transferred to the citizen's fitness card. The designated cooperative sports venues expanded from the original 27 to 47. In 2014 alone, there were 6,163 people in Suzhou applying for Sunshine Cards. According to statistics, the average weekly consumption of card users reaches approximately 17,000 (19). In 2017, the Department of Human Resources and Social Security of Jiangsu Province has stopped the medical insurance transfer business and ended the form of economic binding between medical insurance and gymnasiums. Residents of Suzhou City can apply for Sunshine Fitness Card separately according to their needs, because Sunshine Fitness Card is very popular locally. This has played a positive role in guiding the public to exercise and fitness, improving the overall health of the whole people, and alleviating the pressure on local medical treatment. It is a beneficial attempt to ISM.

In 2016, with “Sunshine Fitness Card” as the carrier, the local government effectively integrated the sports and fitness venues with different business models, and built the “Sunshine Fitness Card” network service system throughout the city. The national fitness service covers the whole urban area of Suzhou, which basically meets the needs of citizens for nearby and convenient participation in fitness. As of June 30, 2020, Sunshine Fitness Card has a total of 79 designated venues, including swimming, fitness, badminton, basketball, football, physical test and other events.

The Suzhou Model is feasible for local sports departments and medical institutions to cooperate in the form of an all-in-one card. However, since the implementation of the “Sunshine Fitness Card” policy, there have been the following shortcomings:

(1) The depth of ISM is not enough: the practice of guiding participants to perform scientific exercises is relatively weak, and there is a lack of regular physical examination feedback and adjustment exercise plans for cardholders.

(2) Fitness facilities lack professional medical services: Fitness facilities do not provide physical fitness tests and effective guidance based on the health status of cardholders, thus turning fitness venues into “sports rehabilitation rooms” of medical departments, realizing proper integration of “sports” and “medical care.”

### Shanghai Model: “1+1+1” community active health project in Jiading District

In response to the strategy of building a healthy China, the Shanghai Municipal Bureau of Sports hosted the first Community (Shanghai) Sports Forum in 2016, the theme of which was “Changes in the Development and Construction of the Community Sports Organizations” (20). The forum put forward specific requirements in terms of physical fitness and medical services and introduced a “Community Active Health Plan.” Jiading District, located in the northwest of Shanghai, took the lead in proposing the community ISM work model of which its main purpose is to: promote the ISM community physical test station and to promote non-medical health interventions. Furthermore, to take community comprehensive prevention and treatment methods for chronic disease patients, to receive scientific medical and health guidance, and relieve the condition through sports.

One of the measures is to incorporate health promotion into community medical services and build a “1+1+1” community work team, namely: one resident self-management team leader (recommended by patients), one community doctor and one social sports instructor. Among them, community family doctors provide medical consultation for patients; social sports instructors are responsible for teaching various fitness exercises and providing exercise rehabilitation guidance to patients with chronic diseases such as diabetes, and hypertension. Establish physical fitness monitoring centers in community streets to provide monitoring services. According to the inspection results, instruct community residents to carry out targeted exercises to improve the overall health level within the range of the community. In 2016, the district government issued the implementation plan of residents' fitness in Jiading District (2016-2020) (21). This has enabled the “1+1+1” community active health project to move toward an institutionalized and standardized operation track and promote the goal of building a healthy China. At present, the overall level of Jiading's national fitness is at the fore-front of Shanghai (22).

However, the “1+1+1” Community Active Health Project in Shanghai has also exposed the lack of enthusiasm interaction between sports and the medical system, and management obstacles in implementing the specific services of ISM. In the actual operation process, it relies too much on the government's health protection service function and often needs to rely on neighborhood committees or streets to coordinate the work between sports and health departments, such as physical fitness monitoring, health promotion, business division, capital investment, and benefit distribution.

At present, some areas in China have made active attempts to provide health services based on the ISM. From the perspective of horizontal comparative analysis, the unique development mode of the ISM appears in various regions, which needs to be expressed and realized by explicit forms. For example, Suzhou “sunshine fitness card” policy and Shanghai “1+1+1” community active health project, which reflects the regional nature of the ISM and the specific development mode of the ISM should flexibly adapt to the regional economy, culture, and community sports development status. The ISM mode has not formed a fixed content and form, and there is no general health service mode. Thus, sports and health departments can actively cooperate with each other in health service projects, which is an important link to promote the realization of the ISM.

## Actionable recommendation

In developing the ISM policy, we recommend the following based on available and relevant evidence.

### Health service talent team building

The ISM service system involves knowledge in sports, medicine, nutrition, and health management. To ensure high-quality service, it is necessary to establish a “sports-medicine” talent structure and recruit professionals to form a knowledge management team (23). At the policy level, the Several Opinions of the State Council on Promoting the Development of the Health Service Industry pointed out that “guide relevant universities to reasonably determine the scale of training relevant professional talents… standardize and accelerate the training rehabilitation therapists, health managers, fitness coaches, social sports instructors and other practitioners” (24). The “Healthy China 2030” Plan Outline mentioned: “Cultivate health education teachers and incorporate health education into the content of pre-service and post-service training for physical education teachers” (6). Under the guidance of the policy, colleges and universities, especially sports and medical colleges, can jointly set up professional courses and practical teaching system featuring ISM to cultivate comprehensive talents. The government should give special funds and facility support to the pilot universities to promote the training of ISM professionals. In addition, the government should establish a continuing education and vocational certification system of ISM to improve practitioners' professional recognition.

### Strengthen the promotion and increase the publicity of ISM

Although Chinese residents have improved their awareness of health promotion and rehabilitation, they have not yet fundamentally understood the connotation of ISM. At the initial stage of the ISM trial, the service targets were mainly urban residents. The community is the main place for the spread of health concepts and the provision of ISM services. Thus, the promotion of the health concept of ISM in urban communities should be strengthened. For example: to strengthen the health risk awareness of adolescents, cultivate their good lifestyles and lifelong exercise habits; conduct publicity and education on disease prevention and scientific exercises to residents in community service centers, and provide health consultations to promote residents to establish a correct outlook on exercise and health; make full use of the media, newspapers, and the Internet to promote the health value of ISM to the society.

### Practice ISM based on regional characteristics

The development of ISM in China is in its infancy and has not yet formed a fixed content and form. Sports and medical management departments should actively cooperate, formulate guiding opinions and policies, and do reasonable coordination work in terms of business division and benefit distribution. The health service projects in which the sports and medical departments can cooperate in the region should be selected according to the actual situation to meet the health service demands of residents in various regions. For example, the State General Administration of Sports and Beijing TV Sports Club jointly planned a pilot program called “Moving for Pregnancy” (25). This program is recommended by experts from Beijing Tiantan Hospital to 100 obese patients with polycystic ovary infertility to receive a 28-day exercise weight loss course at the Fitness Center of the Training Bureau of the State Sports General Administration. Each class is 1.5 h and 2 to 3 classes per week. Changzhou Sports Hospital (Jiangsu, China) screens people with sub-health and chronic diseases through physical examinations and sets up a special health consultation room in the Sports and Health Promotion Center to facilitate patients to understand the specific content of the ISM (26). Although the exact form of practice has not been formed, the exploration of promoting the ISM in some provinces and cities undoubtedly provides a useful reference for the ISM into a scientific and reasonable development track.

## Conclusion

The ISM policy meets people's needs for sports, medical treatment and health at the community and family levels, and has become a new health service model for the public. ISM embodies the mutual complement and promotion of sports and medicine. The implementation of sports training for health promotion is an appropriate choice in response to the ISM policy. The “Suzhou Model” and the “Shanghai Model” are valuable attempts to implement the policy of ISM, laying the foundation for the combination of “Sports” and “Medicine” in the future. At present, the coordinated development of ISM in China is still in its infancy, and there are still some shortcomings in the implementation of the ISM model. Research on the model still requires the cooperation of the government, society, schools, and others including private sectors to promote the long-term operation of the development model, to realize the function and effect of ISM under the new situation.

## Author contributions

SG and LD conceived the manuscript and revised drafts. TZ and ZN wrote the first draft. All authors contributed to the article and approved the submitted version.
